# Changes in the Cystic Fibrosis Airway Microbiome in Response to CFTR Modulator Therapy

**DOI:** 10.3389/fcimb.2021.548613

**Published:** 2021-03-17

**Authors:** Buqing Yi, Alexander H. Dalpke, Sébastien Boutin

**Affiliations:** ^1^ Medical Faculty, Institute of Medical Microbiology and Virology, Technische Universität Dresden, Dresden, Germany; ^2^ Department of Infectious Diseases, Medical Microbiology and Hygiene, University Hospital Heidelberg, Heidelberg, Germany; ^3^ Translational Lung Research Center Heidelberg (TLRC), German Center for Lung Research (DZL), University Hospital Heidelberg, Heidelberg, Germany

**Keywords:** cystic fibrosis, CFTR modulator therapy, host–bacteria interaction, *Pseudomonas aeruginosa*, airway microbiome

## Abstract

The development of CFTR modulator therapies significantly changed the treatment scheme of people with cystic fibrosis. However, CFTR modulator therapy is still a life-long treatment, which is not able to correct the genetic defect and cure the disease. Therefore, it becomes crucial to understand the effects of such modulation of CFTR function on the airway physiology, especially on airway infections and inflammation that are currently the major life-limiting factors in people with cystic fibrosis. In this context, understanding the dynamics of airway microbiome changes in response to modulator therapy plays an essential role in developing strategies for managing airway infections. Whether and how the newly available therapies affect the airway microbiome is still at the beginning of being deciphered. We present here a brief review summarizing the latest information about microbiome alterations in light of modern cystic fibrosis modulator therapy.

## Introduction

Cystic fibrosis (CF) is the most common genetic disorder among Caucasian populations (Petrova and Sauer, 2009). It is primarily caused by mutations in the *CFTR* (cystic fibrosis transmembrane regulator) gene that encodes a cAMP-dependent chloride and bicarbonate channel (Vankeerberghen et al., 2002; Goss and Burns, 2007). Over 2100 variants have been characterized to date (De Boeck et al., 2014; Farrell et al., 2017), which can be grouped into 6 classes, and these mutations cause various functional defects of the CFTR protein [reviewed in (Grasemann, 2017; Burgener and Moss, 2018). Although CFTR dysfunction results in abnormalities in all exocrine glands, the pathophysiological changes occurring in the lung are currently the most life-limiting complications. Dysfunction in the chloride channel causes mucus hypersecretion and impaired mucociliary clearance. Static airway mucus then favors microbial colonization, leading to increased stimulation of the immune system, and resulting in chronic lung inflammation (Kreda et al., 2012; Mall and Hartl, 2014; Elborn, 2016). The hyperactive inflammatory responses contribute to a decline in lung function and eventually lung failure (Gellatly and Hancock, 2013). CF pathology is therefore characterized by the progressive loss of lung function through a cycle of infection, inflammation, and tissue damage. It is noteworthy that there is evidence from animal models that impaired mucociliary clearance due to CFTR dysfunction may lead to lung disease and inflammation even in the absence of infection (Rosen et al., 2018).

Since infection plays a critical role in CF pathology, many studies have been performed to investigate the airway microbial community structure in people with CF. In the past few years, the cost-reduction of next-generation-sequencing (NGS) has made it possible to apply culture-independent methods to investigate the CF airway microbiome. Multiple studies have revealed diverse polymicrobial communities in CF respiratory samples (Zhao et al., 2012; Boutin et al., 2015), and identified many previously undescribed or uncultivable bacteria (Zhao et al., 2012; Boutin et al., 2015; Cuthbertson et al., 2016; Zemanick et al., 2017; Acosta et al., 2018). The airway microbiome was also studied in healthy subjects mainly with bronchoscopically acquired specimens or sputum samples. It has been shown that lower airway microbial communities are similar to oropharyngeal communities (Morris et al., 2013; Dickson et al., 2015; Dickson et al., 2017; Man et al., 2017). Studies comparing healthy individuals with patients with chronic respiratory diseases showed that the airway microbiome is more diverse in healthy people (Erb-Downward et al., 2011; Zemanick et al., 2017). One of the first findings in CF microbiome studies was the correlation between the reduction of bacterial diversity with increasing age, antibiotic use, lung function decrease, and disease progression (Cox et al., 2010; Zhao et al., 2012; Coburn et al., 2015). The decline of diversity was often linked to the dominance of one typical CF pathogen within the microbiota such as *Pseudomonas aeruginosa or Staphylococcus aureus*.

Studies in infants with CF showed that the airway microbiota becomes rich in oropharyngeal taxa at 1-2 years of age. In 3-5 year-old subjects, one or a few CF pathogens often come to dominate the airway microbial community (Muhlebach et al., 2018; Cystic Fibrosis Foundation, 2019). In adolescents and adults, prior studies have shown that the airway microbiome can be either polymicrobial showing a “healthy like” microbiome characterized by the prevalence of oral commensal bacteria, or mono-specific with the dominance of a typical CF pathogen. In all these studies, samples dominated by one typical CF pathogen (e.g. from the genera *Pseudomonas*, *Staphylococcus*, *Stenotrophomonas*, or *Burkholderia)* were associated with decreased microbial diversity and positively correlated to worsened lung functions, higher inflammation, and increase in antibiotic exposure (Klepac-Ceraj et al., 2010; Van Der Gast et al., 2011; Brown et al., 2014; Boutin et al., 2015; Coburn et al., 2015; Flight et al., 2015; Zemanick et al., 2017). It remains unclear how to prevent the decrease of bacterial diversity in people with CF at an early stage. More studies, especially with the metagenomics approach, are needed to investigate the complicated interactions between pathogenic and commensal bacteria in the CF microbiome community. It also should be pointed out that most studies on the CF microbiome have so far focused on the bacterial fraction, but fungi and viruses exist in CF respiratory specimens as well and need more focus in the future (Kim et al., 2015; Kramer et al., 2015).

Previously, CF therapies were only able to address symptoms, not the disease itself, whereas the recently developed CFTR modulator therapies directly address the underlying mutation. By studying the clinical effects of CFTR modulator therapy and its impact on the airway microbiome, we will better understand the interplay between CFTR function, the pathogenesis of CF lung disease, and airway infections. This integrative knowledge will presumably lead to more sustainable treatment strategies for controlling chronic CF airway infections.

## CFTR Modulator Therapies

The initial therapeutic milestone for drug development addressing the causative CFTR function defect was the approval of ivacaftor for clinical use in patients with G551D, a mutation resulting in defective channel opening. Functionally, ivacaftor is considered a potentiator, being able to increase the function of a residual protein. The FDA recently approved an extended label of ivacaftor to many rare and ultra-rare *CFTR* mutations with gating defect or residual function. For the majority of patients with CFTR protein folding defects, no effects were observed (Flume et al., 2012; Grasemann, 2017). Next, the development of correctors that may improve the defective protein trafficking was another important achievement. Although using a corrector as monotherapy was only of modest effect compared with that of using a potentiator as monotherapy, the combination of a potentiator and a corrector showed beneficial clinical effects (Clancy et al., 2012; Boyle et al., 2014). Rescue of the CFTR trafficking defect by correctors (lumacaftor or tezacaftor) in combination with a potentiator stimulating channel gating has been proved effective in patients with F508del, a mutation causing protein folding defect. However, the fixed combination of lumacaftor–ivacaftor has raised concerns about side effects (Burgel et al., 2020). Besides, this therapy is only approved for F508del homozygote treatment. Later, clinical trials of dual-combination tezacaftor-ivacaftor therapy demonstrated a significant improvement in lung functions in patients with either homozygous or heterozygous F508del mutation, with effects similar to that of lumacaftor–ivacaftor but with a better side-effect profile (Burgener and Moss, 2018). With the FDA approval of using CFTR modulator Trikafta (elexacaftor, tezacaftor, and ivacaftor as a combination drug) for treatment of CF in patients >=12 years old, there are finally effective treatment options available for most *CFTR* mutation types (Voelker, 2019).

However, CFTR directed treatment are still lacking for many specific *CFTR* mutations. Importantly, the CFTR modulator therapy cannot cure the disease and remains a life-long treatment. Furthermore, except ivacaftor therapy and lumacaftor-ivacaftor therapy, other types of modulator therapies have not been approved for use in individuals younger than 6 years of age. Owing to these limits, to achieve the best treatment effect and realize the long-term goal of developing a cure for this disease, it is important to keep investigating all critical aspects implicated in the pathophysiology of CF, among which understanding the dynamics of airway microbiome of people with CF in response to various treatments is essential for developing strategies to manage chronic CF airway infections (Cystic Fibrosis Foundation, 2020).

## How CFTR Modulator Therapies Affect the Airway Microbiome

Antibiotic treatment is widely used in CF treatment to control respiratory infection, the effect of which is however limited by many factors, such as the resistome of the CF microbial community (Allemann et al., 2019). Interestingly, *in vitro* data have shown that some CFTR modulators have direct antimicrobial activities (Davies and Martin, 2018). For example, ivacaftor has been shown to have quinolone-like anti-infective activities against *S. aureus* in a dose-dependent fashion, while its effect on *P. aeruginosa* is much weaker (Reznikov et al., 2014). In addition to direct antibacterial activities imposed by antibiotics or other molecules, bacterial community structure can also be regulated by the local microenvironment (Héry-Arnaud et al., 2019). Therefore, any treatment that can change the local microenvironment, can theoretically alter the bacterial community structure as well. By correcting the dysfunction in the chloride channel, CFTR modulator therapy may improve mucociliary clearance and reduce static airway mucus (Mall et al., 2020). Since static airway mucus is one of the major triggers for chronic infection and inflammation in CF, it is expected that by decreasing airway mucus, CFTR modulator therapy may reduce the bacterial burden of typical CF pathogens, such as *P. aeruginosa*, and alleviate chronic infections in people with CF. Furthermore, it has been shown that CFTR modulators could affect inflammatory cells and inhibit the production of pro-inflammatory cytokine IL-18 (Jarosz-Griffiths et al., 2020). The potent anti-inflammatory properties of CF modulators might also have an impact on the airway microbiome.

Investigations about the effects of CFTR modulators on the airway microbiome have so far focused on single-modulator ivacaftor treatment for patients with at least one G551D allele, and only one recent study investigated the effects of lumacaftor-ivacaftor on airway microbiome in F508del-homozygous individuals. In **Table 1**, we have summarized the studies addressing the effects of modulator therapies on microbial features (Rowe et al., 2014; Bernarde et al., 2015; Heltshe et al., 2015; Hisert et al., 2017; Peleg et al., 2018; Ronan et al., 2018; Harris et al., 2019; Einarsson et al., 2021; Graeber et al., 2021). Effects of ivacaftor therapy on *P. aeruginosa* sputum level and other clinical parameters have been assessed in 133 patients of age 6 and older in a longitudinal cohort study (Rowe et al., 2014). In addition to highly improved clinical outcomes such as decreased sweat chloride and enhanced lung function, a reduction of *P. aeruginosa* culture positivity has been observed after 6-month therapy, and the reduction was more profound in patients with less established disease (Heltshe et al., 2015). The results of subsequent investigations indicated that CFTR modulator ivacaftor therapy may lead to evident reductions in sputum *P. aeruginosa* density and airway inflammation, and reductions in the relative abundance of *P. aeruginosa* mostly co-occurred with increases in the diversity of the bacterial community, and with reciprocal increases in the relative abundance of commensal bacteria such as *Streptococcus*, *Prevotella*, *Veillonella* and other taxa (Hisert et al., 2017). Two studies reported no significant changes in global microbial composition in sputum microbiota after six-month or 1-year ivacaftor treatment. Since one of the studies (Bernarde et al., 2015) only included three subjects, it is difficult to detect any significant changes due to a lack of statistical power. The other study (Harris et al., 2019) included 31 subjects, but it is noteworthy that in this study patients who were able to expectorate sputum before ivacaftor treatment but stopped expectorating after 6-month therapy were excluded, which might to a certain extent lead to missing of subjects experiencing substantive improvement in their disease course. Also, it has been shown that changes in antibiotic exposure, taken during modulator therapy to treat infections, were associated with changes in sputum microbiota composition (Peleg et al., 2018). The results of one study with 20 patients aged 18-65 indicated that antibiotic exposure had a stronger effect on microbial communities in sputum samples compared to the effect of ivacaftor modulator therapy. Only in subjects without changes in antibiotic exposure, ivacaftor treatment was associated with a reduction in total bacterial load. Here a change in antibiotic exposure was defined as any escalation or de-escalation in antibiotic use in the two weeks prior to sputum sample collection (Peleg et al., 2018). In another one-year study with 14 patients, with a combination of culture and culture-independent approaches, it has been shown that microbial community richness and diversity of sputum microbiota increased after ivacaftor treatment, which was correlated with lower levels of the main markers of inflammation (Ronan et al., 2018; Einarsson et al., 2021). Interestingly, the relative abundance of streptococci increased, while the relative abundance of *Pseudomonas* spp. decreased (Ronan et al., 2018). However, it needs to be pointed out that while the relative abundance of Streptococci increased, their absolute abundances remained unchanged, as shown in the study from Hisert et al. (Hisert et al., 2017), suggesting ivacaftor therapy did not result in increased absolute abundance of this taxon, and it is so far unclear if these changes in relative abundances of commensal bacteria correlate with improved clinical or inflammatory measures. It is also noteworthy that although the ivacaftor therapy resulted in a reduction of sputum *P. aeruginosa* in several studies, it rarely eradicated *P. aeruginosa* infection. It has been shown that the decreased sputum loads of *P. aeruginosa* after ivacaftor therapy rebounded later in most samples (Hisert et al., 2017). This phenomenon suggests other measures for controlling infection should be applied in parallel to reach the best treatment effect (Saiman, 2019). Besides, although fungi and viruses exist in CF respiratory specimens, the effect of modulator therapy on them is unknown. Effects of lumacaftor-ivacaftor therapy on sputum microbiome have been recently assessed in 14 patients aged 12-41 with homozygous F508del. A significant reduction of total bacterial load and an increase of alpha diversity of the sputum microbiome have been observed (Graeber et al., 2021), which is largely consistent with the reported effects of ivacaftor on airway microbiome in patients with at least one G551D allele.

**Table 1:** Effects of CFTR modulator therapies on microbial features.

Author	Study location	Sample type	Microbiology method	Subject number	Age	Genetic background	Treatment	Treatment period	Observations
**Rowe et al., 2014**	USA	sputum or oropharyngeal swab lrculture data)	Culture; 16S rRNA sequencing; qPCR	133	6 and older	at least one G551D CFTR allele	ivacaftor	6 months	Reduction of *P. aeruginosa* burden after 6-month therapy.
**Bernarde et al., 2015**	France	sputum	qPCR; 16S rRNA sequencing	3	range 10-20	at least one G551D CFTR allele	ivacaftor	around 1 year	No significant changes in global microbial composition.
**Heltshe et al., 2015**	USA	sputum or oropharyngeal swab (culture data)	Culture	151	6 and older	at least one G551D CFTR allele	ivacaftor	6 months	*P.aeruginosa* culture positivity decreased with 29% patients shifting from *P. aeruginosa* positive to negative.
**Hisert et al., 2017**	USA	sputum	Culture; 16S rRNA sequencing; qPCR	12	range 22-57	at least one G551D CFTR allele	ivacaftor	> 2 years	Reduction in the relative abundance of *P. aeruginosa* and increases in the relative abundance of commensal bacteria, such as Streptococcus, but no changes in the absolut e abundance of Streptococcus. Sputum *P. aeruginosa* densities decreased first but rebounded later.
**Peleg et al., 2018**	Australia	sputum	qPCR; 16S rRNA sequencing	20	range 18-65	at least one G551D CFTR allele	ivacaftor	4 weeks	Changes in microbiota composition were associated with changes in antibiotic exposure. For subjects without changes in antibiotic exposure, ivacaftor treatment was associated with a reduction in total bacterial load.
**Ronan et al., 2018**	Ireland	sputum	16s rRNA sequencing	14	6 and older	at least one G551D CFTR allele	ivacaftor	1 year	Microbial community richness increased. Relative abundance of commensal bacteria e.g. streptococci increased, while the relative abundance of *Pseudomonas* spp. declined.
**Harris et al., 2019**	USA	sputum	qPCR; 16S rRNA sequencing	31	10 and older	at least one G551D CFTR allele	ivacaftor	6 months	No association between ivacaftor treatment and airway microbial communities was detected.
**Einarsson et al., 2021**	Ireland	sputum	Culture; 16S rRNA sequencing; qPCR	14	6 and older	at least one G551D CFTR allele	ivacaftor	1 year	Microbial community richness and diversity increased, which was correlated with lower levels of circulating inflammatory markers.
**Graeber et al., 2021**	Germany	sputum	16S rRNA sequencing	14	range 12-41	F508del homozygous	lumacaftor- ivacaftor	8-16 weeks	Total bacterial load decreased, while alpha diversity of the airway microbiome increased.

## Discussion

In this minireview, we have summarized the current knowledge about the airway microbiome alterations in light of modern cystic fibrosis modulator therapy. Most studies investigating the effects of CFTR modulator therapies on airway microbiome have reported significant changes in airway microbial composition after treatment, indicating that modulation of CFTR has the potential to alter the microbiome. A reduction in sputum *P. aeruginosa* abundance has been detected in several studies, suggesting a beneficial effect of modulator therapy. However, in a few other studies, no changes in *P. aeruginosa* abundance were observed. This inconsistency of the results might be due to the differences in enrollment number, subject age, treatment period, or detailed investigation protocol among these studies. Besides, although still controversial, some studies demonstrated that certain CF pathogens or their exoproducts may impact the efficacy of modulators (either by reducing or by increasing) (Stanton et al., 2015; Maillé et al., 2017; Gentzsch et al., 2018; Ruffin et al., 2018).

It appears that CFTR function and airway microbiome are interrelated, and correction of the CFTR function defect results in improvement of airway bacterial community structure towards a decrease in *P. aeruginosa* relative abundance and an increase in alpha diversity. However, the underlying mechanisms of how the correction of CFTR function may alter the airway microbiome remain elusive. Improved mucociliary clearance, changes in the biophysical properties of the microenvironment, the antimicrobial and anti-inflammatory properties of CF modulators, or disruption of a vicious cycle may all possibly contribute to changes in the airway microbiome.

The studies we reviewed here only included subjects age 6 years and older, many of whom had already established infections when the modulator therapy started. It is known that the rise of typical CF pathogens with more advanced disease starts at 3-5 years of age, whereas in 1-2 years old individuals, the airway microbiome is usually polymicrobial with a high relative abundance of oral commensals (Muhlebach et al., 2018; Cystic Fibrosis Foundation, 2019). Based on the reported effects of modulators on individuals age 6 years and older, it is reasonable to assume, that if the modulator therapy is initiated prior to the establishment of chronic infections, it might be able to prevent the rise of typical CF pathogens, and therefore prevent the establishment of infections. Thus, infants with CF would likely benefit most from CFTR modulator therapy. Regarding the effects of modulator therapies on airway microbiome in infants with CF, to our knowledge, no clinical data have been reported yet. Longitudinal studies focusing on the pediatric population < 6 years old are needed to provide information about the long-term effects of sustained use of CFTR modulators in children.

Although multiple modulator therapies are now available for the treatment of patients with a wide range of *CFTR* mutations, up to now, the focus of the studies is the effects of ivacaftor therapy on airway microbiome in patients with at least one allele G551D. For the most frequent CF mutation F508del, the patients can be treated with several other modulator therapies, e.g. lumacaftor-ivacaftor therapy, tezacaftor-ivacaftor therapy, and elexacaftor-tezacaftor-ivacaftor therapy. However, only one study of the effects of lumacaftor-ivacaftor on airway microbiome in F508del-homozygous individuals has been reported, and the effects of other modulator therapies on the airway microbiome have not been reported yet. The relevant investigations need to be performed in patients with diverse genetic backgrounds. From the experimental design aspect, it would be informative to investigate the correlations between airway microbiome parameters and clinical (or inflammatory) parameters, the result of which may provide important information for optimizing personal and precision medicine in CF treatment.

Regarding the NGS methods for analyzing the microbiome, up until now only 16s rRNA targeted approaches have been used in the studies investigating the effects of CFTR modulator therapies on airway microbiome, whereas the shotgun metagenomics approach has not been utilized yet. In addition to the advantage of achieving information about gene repertoire of the microbiome, with the shotgun metagenomics approach, it is possible to identify the frequency and abundance of each component in the community at the species or even strain level (Scholz et al., 2016; Franzosa et al., 2018), which cannot be resolved with the 16s rRNA approach. More studies with the shotgun metagenomics approach are needed to provide more accurate information about the inter-microbial interactions within the CF microbial community.

In summary, the results of most prior studies indicate that airway bacterial community structure can be regulated by CFTR modulator therapies, at least to a certain extent. Owing to the highly limited number of studies investigating this topic, this topic is just at the beginning of being deciphered. To reach a more comprehensive understanding, more investigations should be carried out to study the effects of lumacaftor-ivacaftor therapy, tezacaftor-ivacaftor therapy, and elexacaftor-tezacaftor-ivacaftor therapy in patients with diverse genetic backgrounds. Several other aspects need to be addressed as well including the effects of modulator therapy on the airway microbiota in individuals < 6 years old, the correlations between airway microbiome changes and clinical (or inflammatory) parameter alterations, or the inter-microbial interactions within the CF microbial community. Controlling chronic CF airway infections is one of the most important tasks for physicians in the healthcare management of people with CF. In addition to utilizing antibiotics, is it possible to apply other strategies to fight pathogens in the lung, especially *P. aeruginosa*? Understanding the dynamics of the airway microbiome in response to different therapies and unraveling the interaction between pathogens and commensals might be able to provide answers to this important question.

## Author Contributions

BY, AD, and SB performed literature research and wrote the manuscript together. All authors contributed to the article and approved the submitted version.

## Funding

This work was supported in part by the German Ministry for Education and Research (82DZL004A1 to SB) and from the German Cystic Fibrosis Association Mukoviszidose e. V. (Project number 1805 to AD and SB).

## Conflict of Interest

The authors declare that the research was conducted in the absence of any commercial or financial relationships that could be construed as a potential conflict of interest.
